# Association between Diabetes Knowledge and Self-Efficacy in Patients with Type 2 Diabetes Mellitus in China: A Cross-Sectional Study

**DOI:** 10.1155/2020/2393150

**Published:** 2020-09-25

**Authors:** Tieying Qiu, Jin Huang, Weixing Wang

**Affiliations:** Clinical Nursing Teaching and Research Section, The Second Xiangya Hospital, Central South University, Changsha 410010, Hunan Province, China

## Abstract

**Objective:**

This study aimed to explore the association between DM knowledge and self-efficacy in Chinese patients with T2DM. The influence factors for DM knowledge were explored, and evidence on interventions was provided to patients for information.

**Design:**

A cross-sectional survey was conducted in various hospitals in Hunan Province, China, from April 2017 to February 2019, by using multistage stratified randomized sampling. DM knowledge and self-efficacy were measured using the Audit of Diabetes Knowledge (ADKnowl) and the General Self-efficacy (GSE) Scale. The relationship between the ADKnowl and the GSE scores was analyzed using the Spearman correlation analysis. Differences in the ADKnowl and GES scores among groups with different sociodemographic characteristics were computed; significant variables and GES scores were input in the multiple stepwise linear regression model to predict the influencing factors of ADKnowl scores.

**Results:**

A total of 1,512 eligible patients with T2DM were included in this study, and their mean ADKnowl score was 59.04 ± 16.24. The top score of the eight dimensions in the ADKnowl scale was reducing the complication risk by 71.01%. The mean GSE score was 2.42 ± 0.59. The Spearman correlation analysis showed that the GSE score displayed a significantly positive correlation with DM knowledge at the ADKnowl scale (*r* = 0.172, *P* < 0.001). The best fit multivariable linear regression analysis revealed eight variables that explained 37.6% of the variance of ADKnowl scores. They were diabetes-learning experience, educational background, complication, therapy, waist-to-hip ratio, diabetes duration, marital status, and GSE (*R*^2^ = 0.376, *F* = 5.971, *P*=0.015).

**Conclusions:**

In Chinese patients with T2DM, the self-efficacy in managing DM positively influenced DM knowledge. DM knowledge, as a protective factor, conversely improved the efficiency of self-management for T2DM. Some ignored influence factors in previous studies can be showed by investigating and analyzing from two scales. Health educators and promoters should help in developing DM knowledge and self-efficacy.

## 1. Introduction

Diabetes mellitus (DM) is a major public health problem in China and requires attention from policymakers, clinicians, and public health organizations. DM leads to hyperglycemia due to metabolic disorder and is divided into three types, namely, type 1 DM; type 2 DM (T2DM), which accounts for the majority; and gestational DM [1]. Nearly half of Chinese adults in China may have prediabetes [2]. The prevalence rate of DM in 2010 is 11.6% and has risen by 5.04% in 2017 [3]. As a result of China's large population, the number of patients with DM is estimated to reach 142.7 million by 2035 [4]. If the blood glucose management is poor, various complications may occur, further aggravating the disease. Several researchers have found that 76.4% of people with DM suffers from at least one complication [5]. Microvascular and macrovascular complications, including ischemic heart disease, stroke, diabetic foot, neuropathy, nephropathy, and retinopathy, are common in DM patients with poor glucose management [6, 7]. Therefore, effective DM self-management, including controlling blood glucose and preventing complications, is essential for T2DM therapy, and patients should perform DM self-care treatments systematically and scientifically.

The correctness of perception and prevention of the disease are the foundations of the effectiveness of controlling DM. Patients with DM should always take several measures (such as reducing smoking, controlling blood pressure and blood lipids, and keeping physical activities) in addition to controlling blood glucose to prevent complications [8]. Thus, DM patients having good DM knowledge and awareness are a benefit for them to use appropriate methods to solve health problems timely, including a comprehensive understanding of medical guidance on diet and nutrition, physical activity, medication, and lifestyle [9]. However, most studies from developing countries in Asia reported that the patients with T2DM still maintained poor DM knowledge and lack of scientific glycemic control, dietary management, and complication prevention [10–12]. For instance, in Saudi Arabia, only 13.3% of patients with DM demonstrated good knowledge. Poor knowledge is most apparent in response to the questions related to inappropriate food when the blood glucose is low [13]. Islam et al. [14] also found that older DM patients in Bangladesh showed significantly poorer knowledge than the younger age groups because of insufficiency in acquiring new technologies and information, and only 39% older DM patients knew that the regular physical exercises can prevent diabetes. Therefore, it is necessary for DM patients, especially aged patients, to increase their knowledge on DM, thereby increasing the effect of self-management.

Previous studies have found that general self-efficacy (GSE) plays a critical role in DM self-management [15]. The GSE is an individual's overall self-confidence, behavior, thinking, and emotional responses when facing different environmental challenges or new things [16]. Culturing self-management behavior and establishing positive attitude for treatment can improve GSE [17]. The recommended GSE, as an important protective factor for behavioral ability, can alter people's automatic responses and self-behavior [18]. Nevertheless, T2DM patients have paid considerable attention to changing their attitudes and ignored GSE in disease popularization. Seldom large sample studies on the association of GSE with the knowledge of patients with DM in China exist. Enhancing the GSE of patients on the treatment guidelines for T2DM is commonly recognized in the western world [19, 20].

However, it is still unclear about the relation among DM knowledge, GSE, and sociodemographic variables in T2DM patients. Thus, the purpose of our cross-sectional study is to reveal the association between DM knowledge, GSE, and sociodemographic characteristics and to explore the influence factors of DM knowledge in Chinese patients with T2DM.

## 2. Subjects and Methods

### 2.1. Study Design and Patient Selection

This cross-sectional survey was conducted from April 2017 to February 2019 in Hunan Province, China. The inpatients in the endocrinology departments were selected as respondents in this study. Six representative prefectures (Changsha, Hengyang, Zhuzhou, Yueyang, Changde, and Yiyang) were selected on the basis of their geographic location and economic development status within Hunan Province by using the multistage stratified cluster sampling method. Three hospitals at different levels (3A, 3B, and 2A) were chosen randomly from each of the six prefectures. A total of 18 hospitals and 36 departments were selected. Patients were ranked by their registration in the computerized patient record system and selected as samples in accordance with the random number table. The sample account of each hospital was calculated using Pardoe's formula [21]. After the calculation, the sample size was estimated as 50 (catering for 10%∼20% attrition) for each department.

The inclusion criteria of patients were as follows: (1) over 18 years old, (2) diagnosed with T2DM for at least one year, (3) adults with audio–visual ability, and (4) inpatients in Hunan Province. The excluded patients include those (1) suffering from any mental illness or mental disorder and (2) with DM and other complex diseases, such as paralysis, or who are not cooperating with the researchers.

In total, 1,800 patients were selected randomly as samples in this study. 1,706 participants answered and returned the questionnaires (response rate 94.8%). 194 questionnaires were excluded because of incomplete items (*n* = 87), inconsistent information of patients (*n* = 49), and choosing the same options as the questionnaire (*n* = 58), and 1,512 questionnaires were analyzed in this study (88.6% valid rate).

### 2.2. Data Collection

Before the formal survey, each hospital selected a DM health educator as an investigator. The inclusion criteria of each investigator were as follows: (1) working for at least five years in the endocrinology department, (2) willing to participate in this study, and (3) experience of scientific research and good communication skills. Eighteen selected investigators participated in the face-to-face training lasting for two days and recognized the purpose, method, and research process of this study in the Second Xiangya Hospital of Central South University. Only after completing the training and being qualified, the investigators can begin the research in their hospitals.

Before this survey, every participant should sign and upload the informed consent after well realizing the details of survey. When the investigators confirmed their signatures, the survey began in WeChat group of patients. The questionnaire survey was prepared in accordance with the Wenjuanxin platform (http://www.wjx.cn), a large online survey platform in China. The survey items, as the electronic questionnaire, were sent to each patient. This study was approved by the Second Xiangya Hospital Ethical Committee (Grant No: (2016)S058).

### 2.3. Assessed Variables

The clinical demographic characteristics (i.e., age, gender, educational background, body mass index (BMI), DM duration, complications, therapy, and history of DM courses) were collected. In accordance with the age classification criteria reported by the World Health Organization in 2012, the patients were divided into four age groups, namely, <44, 45–59, 60–74, and ≥75 years [22]. For the smoking and drinking criteria, the patients were divided into smokers and nonsmokers and drinkers and nondrinkers, respectively. The waist-hip ratio (WHR) of patients, measured twice to acquire an average, was calculated as the ratio of each participant's waist circumference to their hip circumference by investigators. Blood was drawn for glycated haemoglobin A1C (HbA1c), and WHR was tested by nurses in the first hospitalizing day.

In this study, the questionnaires were created in reference to the Audit of Diabetes Knowledge (ADKnowl), which was developed in 2001 by Professor Speight of the University of London [23] and revised in 2003. The Chinese version of ADKnowl, which has a content validity index, reliability, and Cronbach's *α* of 0.92, 0.99, and 0.90, respectively, is first translated and revised by scholar Hu [24]. The ADKnowl consists of 26 item sets (111 items), which are related to treatment, sick days, hypoglycaemia, effects of physical activity, reducing complication risks, smoking/alcohol effects, foot care, and diet and food [25]. Three options, namely, “agree,” “disagree,” and “unknown,” were available in the scale for the respondents. The same options in various questions were assigned different values.

The self-efficacy of patients relatively represents DM management. The patients' self-efficacy was measured using the GSE scale with 10 items. Cronbach's *α* was 0.87, and the cumulative variance contribution rate was 77.0%, which indicated positive reliability and validity [26]. The responses for each item were rated using a four-point Likert scale (1, totally disagree; 2, somewhat disagree; 3, somewhat agree; and 4, totally agree). The total score ranged from 10 to 40, and a high score indicated a high self-efficacy level.

### 2.4. Statistical Analyses

All data were analyzed using the SPSS (ver. 22.0; IBM Corp, Armonk, NY, USA). Continuous variables were reported as mean ± standard deviation, and categorical variables were presented as frequencies (%). The association of the demographic variables with the ADKnowl and the GSE scores was assessed using the *t*-test and analysis of variance. The relationship between the ADKnowl and the GSE scores was analyzed using the Spearman correlation analysis. If the *P* value was <0.05 in above tests, significant variables and GSE scores were input in the multivariable linear regression analysis model as independent variables to predict the ADKnowl score. *P* < 0.05 was considered statistically significant.

## 3. Results

### 3.1. Respondent Characteristics

A total of 1,512 subjects (801 (53.0%) males and 711 (47.0%) females) with ages ranging from 45 years to 74 years were included in this study. The subjects had a mean DM duration of 9.31 ± 6.93 years and mean HbA1c of 8.84% ± 2.37%. Most (90.9%) of the patients had T2DM complications. The patients were treated with three independent treatments for DM, namely, oral hypoglycemic agent (OHA) only (492, 32.5%), insulin only (454, 32%), and OHA + insulin (566, 37.5%). The BMI of most patients was ≥24 (801, 53.2%), describing obesity, and 38.9% of the population had a WHR higher than 0.9 (Table 1).

### 3.2. Patients' DM Knowledge and Self-Efficacy

The total ADKnowl score was 59.04 ± 16.24, and the correct answer rate was 54.35%. The ADKnowl scale consisted of eight dimensions, namely, diet and food, treatment, sick days, foot care, physical activity, smoking/alcohol effects, complication prevention, and hypoglycaemia. The ADKnowl scores of each dimension are shown in Table 2. The levels of knowledge in seven domains were low (<60%), while in terms of reducing complication prevention was 71.01%. The mean GSE score was 2.42 ± 0.59, which was in the middle level (Table 2).

### 3.3. Correlation between the GSE and the ADKnowl Scores

The Spearman correlation analysis revealed a significant correlation between the GSE and the ADKnowl scores for each dimension (*P* < 0.001, Table 3). Significant positive and moderate correlations existed between the GSE score and all dimensions of ADKnowl (*r* = 0.121, 0.102, 0.054, 0.146, 0.077, 0.119, 0.163, and 0.091; *P* < 0.001), indicating that patients with high GSE exhibited a high ADKnowl score.

### 3.4. Differences in the GSE and ADKnowl Scores among Various Sociodemographic Characteristics

The association of demographic variables with the ADKnowl and the GSE scores (Table 4) demonstrated a statistically significant correlation between the gender and ADKnowl score, as shown by the higher ADKnowl score of males than that of females (*P* < 0.001). The ADKnowl score of patients aged 45–59 years was significantly higher than those of the other age groups (*P* < 0.001). The ADKnowl scores of those who had undergraduate education and above was the highest. Intelligent and retired patients had higher ADKnowl score than the others (*P* < 0.001). Patients who have complications also had a higher ADKnowl score than the others (*P* < 0.001). Patients using OHA and insulin therapies had a better ADKnowl score than the others, as shown by the gradual increase in the ADKnowl score with increasing DM duration and WHR (*P* < 0.001). Patients with better HbA1c status also had a higher ADKnowl score than the others.

In terms of the GSE score, patients aged below 44 years and who were married had the highest GSE score than the other groups (*P* < 0.05). The GSE score increased with increased level of education. Patients with occupation requiring high intelligence had a higher GSE score than others (*P* < 0.001). People receiving DM education also had better GSE than others (*P* < 0.05). The GSE score showed a negative association with complication and WHR (*P* < 0.05).

### 3.5. Analysis in Influence Factors of the ADKnowl Score

To compare the influence factors of ADKnowl scores, the total ADKnowl score was a dependent variable *Y*. The sociodemographic variables that had significant correlation with the ADKnowl score and GSE score were the independent variables *X* (i.e., gender, age, marriage, education, occupation, smoking habit, drinking habit, history of DM courses, complication, therapy, DM duration, WHR, HbA1c, and total GSE score), and several variables were assigned values (Table 5). Performing the multivariable linear regression analysis, the ADKnowl score, involvement in diabetes-learning experience, educational background, complication, therapy, waist-to-hip ratio, diabetes duration, marital status, and GSE score were predictive factors. All factors presented a positive correlation with the ADKnowl score of T2DM patients (*R*^2^ = 0.372, *F* = 5.971, *P* < 0.05, Table 6).

## 4. Discussion

As is known, the scientific management of T2DM requires adjusted lifestyle, regular glycemic monitoring, periodic screening for complications, and proper pharmacological treatment [27]. It was evident from the literature that DM knowledge was continuously increasing, which might contribute to provide better insight for the development of preventive strategies and management [28]. This research based on a cross-sectional study was conducted to demonstrate the DM knowledge and identify associated factors of T2DM patients in China. DM knowledge was categorized into eight distinct aspects according to self-management of disease in the ADKnowl scale; moreover, patients with good DM knowledge were significantly associated with self-efficacy.

Sufficient knowledge of DM was a prerequisite for appropriate self-management [29]. In this study, only 54.35% T2DM patients had good DM knowledge, demonstrating that the T2DM patients in China faced on an insufficient understanding of this disease. This finding was better than that in the study conducted by Yang et al., in which only 37.2% patients had a clear understanding of DM knowledge in Shanghai [30]. Several descriptive studies found that DM knowledge deficits (especially regarding hypoglycaemia recognition) and inappropriate practices were prevalent among T2DM patients [31, 32]. There were prominent deficiencies in the patients' general DM knowledge in the present study, pertaining to treatment, diet, sick day, and hypoglycaemia management. Diabetes patients would adhere to their medication schedules more closely if they believe in medication efficacy and perceive their illness as manageable [33, 34]. The effect of specific food was essential for controlling blood glucose, as well as their complications. Improper management of hypoglycaemia resulting from unawareness could occur if patients were unable to identify the symptoms of hypoglycaemia and take intervention timely [29]. Knowledge of management of blood glucose levels during sick days was crucial to prevent complications of dehydration and hyperglycaemic crisis. Therefore, acquiring DM knowledge played a critical role in optimizing the glycemic control of patients with T2DM.

Moreover, self-efficacy was a powerful predictor of glycemic control. Self-efficacy, an optimistic factor, increases self-confidence and decreases the patients' stress from disease [35]. In addition, self-efficacy was the specific and dynamic behavior, which focused on beliefs about personal abilities in a specific setting or with a particular behavior, such as dieting or exercise [36]. In this study, the self-efficacy of T2DM patients was 2.42 ± 0.59, remaining a moderate level. Some previous studies showed that educational interventions for patients are known to be effective in increasing self-efficacy, which is vital to influence the adequacy of patients' self-management [37, 38]. Self-efficacy was positively correlated with reducing complication risk, improving foot care, and developing diet or food plan in DM knowledge in our study (*r* = 0.163, 0.146, 0.121; *P* ≤ 0.001, respectively), demonstrating that patients with high GSE scores paid more attention to acquire related knowledge and to positively manage DM. Thus, we suggested devising strategies to improve GSE that would help patients increase DM knowledge and develop a customized diabetes health plan.

As other predictors of DM knowledge in T2DM patients, DM duration, educational background, diabetes-learning experience, and complication were identified. Earlier studies suggested that college educated patients had the best knowledge of DM among whole patients. The researchers speculated that patients with high educational background intent to acquire additional DM knowledge [39]. Our survey demonstrated the systematic DM knowledge with prolongation of DM duration and accumulation of diabetes-learning experience, resulting from further opportunities to acquire knowledge in hospitals or communities. Furthermore, patients suffering from different complications and living with an unsatisfactory situation preferred to achieve DM knowledge. Several researchers showed that 66.7% of T2DM patients with renal complications had improved knowledge. Comparing with the front fact, the percent of patients suffering neuropathy complications was 47%–65% [40].

Additionally, WHR, therapy, and marital status were related with patients' DM knowledge. WHR, a key indicator of central obesity, is an important risk factor of the failure of DM treatment and irregular death [41]. Thus, the population with high WHR should pay further attention to explore related information for preventing and treating DM. This study showed that married, obese, or combination-therapeutic patients always acquired better knowledge because those patients had stabilized social relationship and powerful willing to learn some related methods of self-management. It involved complicated decision making that depends on the patients' perception of their diseases in terms of whether it is controllable, understandable, curable, or serious [42]. Diabetic patients' knowledge of their disease is one of the important determinants of self-management practices. Therefore, analyzing the influencing factors of DM knowledge is very important in diabetes intervention for medical providers and patients.

This study has several limitations. A gap exists between the objective and the actual original data due to the limitations in the sample size of different hospitals, and this gap influences the subsequent analysis to a certain extent. In addition, this survey may likely lead to self-cognitive errors and misunderstanding on certain questions because the responses to the questionnaires are self-reported.

## 5. Conclusion

In this study, DM knowledge is demonstrated to be a remarkable protective factor for DM complications in patients with T2DM via demographic and clinical variable analyses. The patients' self-efficacy is a positive predictor of DM knowledge. The foundation of DM knowledge and self-efficacy of patients with T2DM are established, and a limited consensus is reached through these large-sized questionnaires and analyses. This study provides evidence for further intervention on DM in Central China.

## Figures and Tables

**Table 1:** Demographic and clinical characteristics of patients with type 2 diabetes.

Variables		Frequency	Percentage
Gender, *n* (%)	Men	801	53.0
	Female	711	47.0
Age, *n* (%)	<44	101	6.7
	45∼59	569	37.7
	60∼74	681	45.0
	≥75	161	10.6
Marital status, *n* (%)	Single	32	2.1
	Married	1379	91.2
	Divorced	19 (1.3)	1.3
	Widowed	82	5.4
Educational background, *n* (%)	Primary school and below	407	26.9
	Junior middle school	447	29.6
	Senior middle school	513	33.9
	Undergraduate and above	145	9.4
Occupation, *n* (%)	Intelligence	186	12.3
	Manual	403	26.7
	Retired	768	50.8
	Others	155	10.2
Smoke, *n* (%)	No	1194	79.0
	Yes	318	21.0
Drink, *n* (%)	No	1346	89.0
	Yes	166	11.0
Diabetes-learning experience	Yes	1195	79.0
	No	317	21.0
Complication, *n* (%)	Yes	1375	90.9
	No	137	9.1
Therapy	OHA only	492	32.5
	Insulin only	454	30.0
	OHA + insulin	566	37.4
Diabetes duration (yrs)	<5	448	29.6
	≥5	1064	70.4
BMI (kg/m^2^)	<24	704	46.6
	≥24	801	53.2
WHR	<0.9	426	28.2
	≥0.9	756	50.0
HbA1c (%)	≤9	924	61.1
	>9	588	38.9

**Table 2:** The audit of diabetes knowledge (ADKnowl) scores and the GSES scale.

Variables	Mean ± SD	Maximum	Minimum	Correct rate (%)
ADKnowl scores				
Total	59.04 ± 16.24	98	0	54.35
Diet and food	9.83 ± 3.40	19	0	54.55
Treatment	6.77 ± 3.04	15	0	20.15
Sick days	2.91 ± 1.77	8	0	53.13
Foot care	12.40 ± 4.09	20	0	51.68
Physical activity	4.19 ± 2.36	9	0	57.65
Smoking/Alcohol effects	4.66 ± 2.43	11	0	46.78
Reducing complication risks	11.36 ± 2.56	16	0	71.01
Hypoglycaemia	7.88 ± 2.93	14	0	56.26
GSES scores	2.42 ± 0.59	4	1	

**Table 3:** Spearman correlation between ADKnowl and GSES.

Variables	GSES	
	*r*	*P* value
Total score	0.172	≤0.001
Diet and food	0.121	≤0.001
Treatment	0.102	≤0.001
Sick days	0.054	≤0.001
Foot care	0.146	≤0.001
Physical activity	0.077	≤0.001
Smoking/alcohol effects	0.119	≤0.001
Reducing complication risk	0.163	≤0.001
Hypoglycaemia	0.091	≤0.001

ADKnowl: the audit of diabetes knowledge; GSES: general self-efficacy scale.

**Table 4:** Association between various factors and ADKnowl and GSES.

Variables		ADKnowl (mean ± SD)	*P* value	GSES (mean ± SD)	*P* value
Gender, *n* (%)	Male	60.68 ± 15.69	≤0.001	2.45 ± 0.64	0.174
	Female	57.19 ± 16.65		2.30 ± 0.62	
Age, *n* (%)	<44	59.48 ± 17.33	0.019	2.47 ± 0.74	0.004
	45∼59	60.13 ± 16.36		2.44 ± 0.62	
	60∼74	58.89 ± 15.65		2.33 ± 0.62	
	≥75	55.59 ± 17.17		2.31 ± 0.63	
Marital status, *n* (%)	Single	57.85 ± 18.34	0.001	2.22 ± 0.62	0.038
	Married	59.51 ± 16.02		2.39 ± 0.63	
	Divorced	57.42 ± 15.60		2.25 ± 0.65	
	Widowed	52.00 ± 17.77		2.36 ± 0.59	
Educational background, *n* (%)	Primary school and below	54.85 ± 16.06	≤0.001	2.20 ± 0.56	<0.001
	Junior middle school	59.23 ± 15.98		2.35 ± 0.63	
	Senior middle school	59.99 ± 16.04		2.42 ± 0.64	
	Undergraduate and above	62.98 ± 15.89		2.59 ± 0.64	
Occupation, *n* (%)	Intelligence	64.30 ± 15.04	≤0.001	2.69 ± 0.65	<0.001
	Manual	54.40 ± 17.17		2.30 ± 0.63	
	Retired	60.07 ± 15.84		2.35 ± 0.61	
	Others	59.68 ± 14.15		2.36 ± 0.61	
Smoke, *n* (%)	No	58.60 ± 16.41	0.040	2.35 ± 0.63	0.895
	Yes	60.71 ± 15.49		2.49 ± 0.63	
Drink, *n* (%)	No	58.71 ± 16.41	0.023	2.35 ± 0.63	0.765
	Yes	61.74 ± 14.52		2.59 ± 0.62	
Diabetes-learning experience	Yes	60.53 ± 15.83	≤0.001	2.39 ± 0.65	0.046
	No	53.44 ± 16.55		2.36 ± 0.58	
Complication	No	57.31 ± 16.07	≤0.001	2.39 ± 0.65	0.037
	Yes	61.61 ± 16.45		2.36 ± 0.60	
Therapy	OHA only	55.61 ± 15.95	≤0.001	2.42 ± 0.63	0.202
	Insulin only	59.08 ± 16.11		2.38 ± 0.63	
	OHA + insulin	62.00 ± 16.03		2.35 ± 0.64	
Diabetes duration (yrs)	<5	56.36 ± 16.25	≤0.001	2.38 ± 0.65	0.391
	≥5	60.17 ± 16.11		2.38 ± 0.63	
BMI (kg/m^2^)	<24	58.62 ± 16.10	0.374	2.33 ± 0.62	0.571
	≥24	59.37 ± 16.39		2.42 ± 0.64	
WHR	<0.9	57.35 ± 16.29	≤0.001	2.34 ± 0.60	0.047
	≥0.9	61.09 ± 16.44		2.43 ± 0.66	
HbA1c (%)	≤9	59.70 ± 16.03	0.049	2.39 ± 0.62	0.190
	>9	58.01 ± 16.53		2.36 ± 0.65	

**Table 5:** Assigned values of independent variables.

Variables	Value
Gender	Male = 1, female = 2
Age	<44 = 1, 45∼59 = 2, 60∼74 = 3,≥75 = 4>
Marriage	Single = 1, married = 2, divorced = 3, widowed = 4
Education	Primary school and below = 1, junior middle school = 2, senior middle school = 3, undergraduate and above = 4
Occupation	Intelligence = 1, manual = 2, retired = 3, others = 4
Smoke	No = 1, yes = 2
Drink	No = 1, yes = 2
Diabetes-learning history	No = 1, yes = 2
Complication	No = 1, yes = 2
Therapy	OHA only = 1, insulin only = 2, OHA + insulin = 3
Duration of diabetes	<5 = 1, ≥5 = 2
WHR	<0.9 = 1, ≥0.9 = 2
HbA1c	≤9 = 1, >9 = 2

**Table 6:** Multivariable linear regression analysis of diabetes knowledge.

Variables	*B*	SE	*β*	*t*	*P*	95% CI	
						Lower	Upper
Constant	0.338	5.920		0.057	0.955	−11.282	11.957
Diabetes-learning experience	6.696	1.284	0.163	5.215	<0.001	4.176	9.216
Educational background	2.491	0.476	0.165	5.233	<0.001	1.556	3.425
Complication	5.349	1.069	0.158	5.003	<0.001	3.251	7.448
Therapy	2.848	0.631	0.140	4.511	<0.001	1.609	4.087
WHR	3.129	1.053	0.091	2.970	0.003	1.062	5.196
Diabetes duration	3.799	1.180	0.102	3.219	0.001	1.483	6.116
Marital status	−2.800	1.146	−0.076	−2.444	0.015	−5.048	−0.551
GSE scores	2.872	0.878	0.102	3.272	0.001	1.149	4.596

Overall *R*^2^ = 0.376; model fit, *F* = 5.971; *P*=0.015, statistically significant at *P* < 0.05. *B*, partial regression coefficient for the constant. SE, the standard error around the coefficient for the constant. *β*, standard partial regression coefficient.

## Data Availability

The data used to support the findings of this study are included within the article.
